# Protein expression in human trabecular meshwork: downregulation of RhoGDI by dexamethasone in vitro

**Published:** 2010-02-13

**Authors:** Minbin Yu, Jing Sun, Wei Peng, Ziyan Chen, Xianchai Lin, Xuyang Liu, Mingtao Li, Kaili Wu

**Affiliations:** 1 Zhongshan Ophthalmic Center; State Key Laboratory of Ophthalmology, Sun Yat-sen University, Guangzhou, P.R. China; 2 The Proteomics Lab, Zhongshan School of Medicine, Sun Yat-sen University, Guangzhou, P.R. China; 3 Department of Ophthalmology and Ophthalmic Laboratories, West China Hospital, Sichuan University, Chengdu, Sichuan, P.R. China

## Abstract

**Purpose:**

The characterization of the human trabecular meshwork (TM) proteome is a valuable step toward understanding its role under normal and glaucomatous conditions. This study uses proteomic techniques to investigate the set of proteins expressed in normal human TM and to identify those differentially expressed in response to dexamethasone (DEX) treatment of TM cells (TMCs) in vitro.

**Methods:**

TM tissue (TMT) was isolated from human donor eyes and pooled. Immortalized human TMCs were cultured with or without DEX. Protein extracts from each were separated by two-dimensional electrophoresis (2-DE). Protein spots in TMT gel were excised, destained, and subjected to in-gel tryptic digestion and identification with matrix-assisted laser desorption/ionization time-of-flight mass spectrometry (MALDI-TOF-MS). To determine those proteins whose expression patterns were affected by glucocorticoids, TMCs were treated with DEX and assayed by 3-(4,5-dimethylthiazol-2-yl)-2,5- diphenyltetrazolium bromide (MTT) dye and 2-DE. A differentially expressed protein, RhoGDI, was validated by both western blotting and immunocytological staining.

**Results:**

The comprehensive protein set included more than 850 protein spots from both the TMT and TMCs, as visualized on 2-DE gel. Two-hundred-and-thirty-five spots were successfully identified in the TMT gel. The functional categories of the identified proteins were mainly comprised of metabolic process, cell adhesion, anti-apoptosis, cell motility, carbohydrate metabolic process, signal transduction, and regulation of transcription. During three days of DEX treatment, TMCs’ proliferation was inhibited in a time- and dose-dependent manner, as evidenced by MTT assay. In the 48 h cultured cell group, RhoGDI expression was reduced, as detected by 2-DE, western blotting, and immunocytological staining. In contrast, the expression of RhoA, a target of RhoGDI, increased in response to DEX treatment.

**Conclusions:**

Using the classic proteomic workflow, the main protein complement of normal human TMT was detected, identified, and categorized. The DEX inhibition of RhoGDI expression in TMCs was evidenced.

## Introduction

Primary open-angle glaucoma (POAG) is one of the most common eye diseases causing progressive asymptomatic loss of bilateral vision and visual field resulting in blindness. Since aqueous humor drains mainly through the trabecular meshwork (TM) into Schlemm’s canal and the episcleral veins, the increased intraocular pressure (IOP) is due to an increased resistance to aqueous humor outflow in the TM, a complex of endothelium-like cells surrounded by the extracellular matrix (ECM) [1-4]. The common eye disease, glaucoma, originates from malfunction of the TM tissue (TMT), and is believed to be responsible for the development of POAG. On the other hand, glucocorticoid-induced glaucoma is similar to that of POAG, both in its clinical presentation and pathological TM changes, including effects on the (ECM), cytoskeleton, gene expression, and cell function [2,5-9]. Therefore, treating cells and animals with glucocorticoids has resulted in the creation of effective in vitro and in vivo models for glaucoma research.

TMT proteins involved in maintaining physiologic and pathological status have been extensively studied. Specific genes and their products (e.g., trabecular meshwork-inducible glucocorticoid response protein/myocilin [10-13], fibronectin [14], optineurin [15], procollagen C-proteinase enhancer 1 (PCOLCE1) [16], cytochrome P450 1B1 [17]) found in TM have been shown to play roles in glaucoma pathogenesis. Another protein family that has been widely studied in TM cells (TMCs) includes the Rho GTPase members, such as RhoA and Rac [4,18-20]. Rho GTPases function in regulating actin cytoskeletal organization, and cell adhesive interactions, as well as influencing cell polarity, morphogenesis, migration, vesicle trafficking, cell cycle progression, and transcriptional activity [4,21]. In addition, Rho GTPase has been demonstrated to influence aqueous humor outflow in TM by regulating the actomyosin assembly, cell adhesive interactions, and expression of ECM proteins and cytokines in TMCs [4,22-25]. The Rho GTPases act as molecular switches by cycling between active guanosine-5’-triphosphate (GTP)-bound and inactive guanosine-5’-diphosphate (GDP)-bound forms. Rho GDP dissociation inhibitor (RhoGDI) negatively regulates Rho GTPase activity by inhibiting GDP dissociation and promoting GTP hydrolysis [26,27]. To our knowledge, the differential expression status of RhoGDI in TMCs has yet to be investigated.

Investigations into the molecular mechanism(s) mediating pathological changes of TM are currently underway using genomic techniques [13,28-30]. In addition, proteome analysis has been used to study the protein expression in TM from normal and glaucomatous eyes. Using sodium dodecyl sulfate-polyacrylamide gel electrophoresis (SDS-PAGE), liquid chromatography electrospray tandem mass spectrometry (LC/MS/MS) and other techniques, Bhattacharya and his colleagues found that cochlin deposits were associated with glaucomatous TM. Their studies also revealed protein expression profiles altered between normal and glaucoma TMT, wherein 52 out of 368 identified proteins were detected exlusively in glaucomatous TM [31]. Using proteomic analysis of dexamethasone (DEX)-treated rats, Shinzato et al. [32] reported that 14 proteins were up- or downregulated consistently in TM. Of these 14, two crystallins (βA3 and αA) were upregulated, and the C-propeptides of type I collagen were downregulated by more than twofold. Although proteomic technologies have high throughput and are sensitive, the comprehensive characterization of the human TM proteome, especially in glaucoma patients, remains a challenge, because of the limited availability of intact tissue.

Instead of using human TMT, previous proteomic studies used TMC lines under varying culture treatments [29,33]. Steely et al. [33] used two-dimensional electrophoresis (2-DE) to separate proteins in a transformed TMC strain (GTM3); 87 primary proteins and 93 variants of these proteins were identified by LC/MS/MS.

To investigate the protein expression profile of human TM and to better understand the mechanisms causing elevated IOP resulting from impaired aqueous humor circulation, we performed 2-DE of TM from normal donors and from a TMC line. Gel patterns corresponding to the global protein expression in normal human TM and the cultured TMC line were obtained. Matrix-assisted laser desorption/ionization time-of-flight mass spectrometry (MALDI-TOF-MS) and bioinformatics analysis was used to make the initial step toward documenting the proteome of human TMT; 235 protein spots on a 2-DE gel of TMT were identified and categorized. Furthermore, by analyzing the gel pattern of DEX-treated TMCs and completing immunoassays, we found that RhoGDI responded to the presence of DEX.

## Methods

### Tissue procurement

Human eyes from 30 normal donors (aged 20–35 years old) were obtained through the Guangdong Province Eye Bank, China and used in this study. Within 12 h after death, TMT was dissected in a ring shape. The possibility of contamination by small amounts of sclera was noted, in accordance with the report by Bhattacharya et al. [31,34]. Harvested TM samples were collected in tubes and stored at −80 °C until use.

### Cell culture

An immortal human trabecular meshwork cell line [35] was cultured in Dulbecco’s modified Eagle’s medium and F12 (DMEM/F12) containing 15% newborn calf serum. TMCs were treated with different levels of DEX (10^−5^, 10^−6^, and 10^−7^ M) added to the medium for one- to three-day durations. Since we hoped to find the early expressed proteins responding to DEX treatment, we used a short-term (i.e. within 3 days) cell culture, as previously reported [36,37]. Meanwhile, besides the 10^−7^ M DEX used in most studies [9,37,38], we also used 10^−6^ and 10^−5^ M DEX to see the more obvious effects on TMCs. At various time points, cell cultures were terminated for further analyses. The cellular changes were measured by phase-contrast microscopy and 3-(4,5-dimethylthiazol-2-yl)-2,5- diphenyltetrazolium bromide (MTT) assay (described below). Soluble proteins extracted from the TMCs were used for subsequent 2-DE and western blot analyses.

### Cell viability assays

The proliferation rate of TMCs was measured using an MTT assay [39]. Each group of TMCs was trypsinized and seeded in 96-well plates with an initial density of 5×10^3^ cells per well. Subsequently, cells were cultured in medium with various concentrations of DEX for 1, 2, or 3 d. Upon color development, the absorbance of each sample was measured using a multiwell scanning spectrophotometer. The absorbance of each experiment was analyzed in five wells, and three independent experiments were performed. Statistic analysis (Student’s t-test) was conducted to compare the differences between DEX-treated and -untreated cells at each time point.

### Immunohistochemical assay

TMCs were cultured on a glass coverslip in 12-well chamber dishes, with or without DEX treatment, as described above. Slide-mounted cells were used for immunostaining of RhoGDI by means of an HRP-DAB Cell and Tissue Staining Kit (Maixin-Bio, Fuzhou, China) according to the manufacturer’s instructions. Briefly, TMCs were fixed in ice-cold acetone for 20 min and cell membranes were permeabilized with 0.1% Triton X-100, followed by quenching of the endogenous peroxidase activity with 3% H_2_O_2_ and blocking non-specific binding with goat serum albumin. Then, cells were incubated with antibodies against RhoGDI (#2564; Cell Signaling Technology, Danvers, MA) at 4 °C, overnight. Cells were then incubated with secondary goat antibodies, and color development was done with DAB reagents. A negative control was run, in which the cells were incubated in PBS without the addition of primary antibodies.

### Protein extraction

Pooled TMT was thoroughly cut into small pieces with a scissor. Then an aliquots-of-lysis buffer (8 mol/l urea, 4% CHAPS, 2% Pharmalyte 3–10; GE Healthcare, Ltd., NJ) was added to the samples, followed by homogenization and sonication on ice. The soluble protein was obtained by centrifugation (at 12,000× g for 30 min at 4 °C). For cultured cells, collection was performed by rinsing with PBS, removal from the flask with a rubber policeman, and pelleting at 500× g centrifugation. After adding an aliquot lysis buffer, cell pellets were sonicated. Insoluble materials were removed by centrifugation (at 12,000× g for 30 min 4 °C). The soluble protein portion was quantified by BCA assay using a protein assay kit (Bio-Rad, Hercules, CA) and stored at −80 °C until the next analyses.

### Two-dimensional gel electrophoresis

Two-dimensional gel electrophoresis was performed using instruments, kits, and the 2-DE protocol from GE Healthcare (GE Healthcare Bio-Sciences AB, Uppsala, Sweden), and according to our previously reported protocols [40], unless otherwise specified. Briefly, the extracted protein solution was precipitated with a 2-D clean-up kit. The pellets were dissolved in rehydration solution to a certain volume, according to the size of the immobilized pH gradient (IPG) strips, and centrifuged at 15,000× g for 10 min to remove insoluble materials. The supernatant was used as the loading sample on the IPG strips.

First-dimensional isoelectric focusing (IEF) was performed using the Ettan IPGphor II unit. IPG strips (18 cm, pH 3–10, linear gradient) were loaded with protein samples and rehydrated overnight. Isoelectric focusing was run at 15 °C, totally focused over 8 kVh. First dimension strips were subjected to the standard reduction and alkylation steps before second-dimension electrophoresis, as follows. The strips were soaked for 15 min in 50 mM Tris-HCl solution (pH 8.8) containing 6 M urea, 30% glycerol, 2% SDS, 2% DTT, and 0.001% bromophenol (w/v). They were then soaked for an additional 15 min in the same solution, except that 2.5% iodoacetamide was substituted for the 2% DTT.

The second-dimensional electrophoresis (SDS–PAGE) was conducted on 10% polyacrylamide gels (PROTEAN^®^ II xi Cell; Bio-Rad) with protein molecular weight markers (Takara Biotech Co. Ltd., Dalian, China). At the end of electrophoresis, gels were stained with hot Coomassie brilliant blue R-350 [41].

After destaining, gels were scanned using an ImageScanner II and analyzed by Melanie software (GE Healthcare). Protein spots were first outlined automatically, and many were further outlined manually, and quantified. The measured molecular weight (Mr) and isoelectric point (pI) values of each protein spot were calculated according to the method introduced by Westermeier and Naven [42].

### Mass spectral analysis and protein identification

MS analysis and protein identification were performed as reported previously [40,43]. Following imaging analysis, protein spots on the large gel run with TMT protein were selected for identification. Spots with relatively higher intensities on 2-DE gels were excised, dried, digested with trypsin (sequencing grade; Promega, WI), and mechanically spotted onto MS plates by an Ettan Spot Handling Workstation (GE Healthcare). The molecular masses of peptides were measured by MALDI-TOF-MS using an Ettan MALDI-TOF Pro mass spectrometer (GE Healthcare). On the basis of peptide-mass matching, peptide peaks obtained from mass mapping were identified using the NCBInr database. All spots were examined for methionine oxidation, for iodoacetamide modification, and, with no-limitation, for pI. The confidence of identification was indicated by the number of matching peptides, and the percent of the amino acid-sequence coverage. Proteins were accepted on the basis of four or more peptides having matched [43].

### Cluster analysis of trabecular meshwork proteins

The categories of identified proteins were analyzed by inputting their accession codes into a bioinformatic databse, which automatically classifies genes based on gene ontology (GO) terms. Before GO-based binning, any identified protein with a GI code was recoded according to gene/RefSeq accession numbers, using the websites UniProt, NCBI, and BLAST.

### Western blotting analysis

Western blotting was conducted according to specifications of the antibody manufacturer and our previous report [40]. Briefly, aliquots of lysates (30 μg of protein equivalent) were subjected to 10% SDS–PAGE under reducing conditions, then blotted onto a PVDF membrane. Antibodies against RhoGDI (#2564) and RhoA (#2117), and a Phototope-HRP western blot kit (all from Cell Signaling Technology) were used for western blotting assay. Densitometric analysis was performed to measure the intensity of the RhoGDI and RhoA bands with the use of the molecular imaging software in the Kodak Image Station 4000MM (Kodak, Rochester, NY). Each experiment was repeated three times.

## Results and discussion

### Protein expression pattern on two-dimensional electrophoresis gels

**Figure 1:**
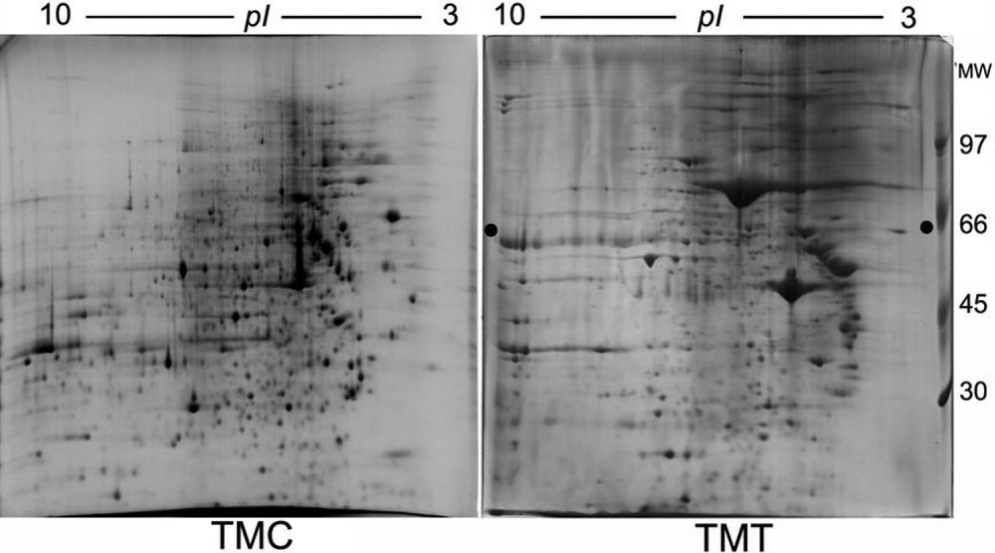
Protein expression map of human trabecular meshwork by two-dimensional gel electrophoreses. Human trabecular meshwork tissue (TMT) and cell (TMC) lysates (approximately 1 mg protein) were applied to 2-DE gel (18 cm strips) that was visualized by Coomassie blue staining.

To date, 2-DE protein-expression, combined gel-spot identification for human TMT has not yet been conducted because of difficulties in obtaining enough proteins from limited tissue samples, although the proteome of TMCs has been extensively studied [29,33,44]. In this study, the 2-DE gel pattern of human TM tissue and a human TMC line was obtained. We first compared the 2-DE protein patterns of human normal TMT to that of TMCs. Figure 1 shows that the protein spot distribution of TMCs is somewhat different from that of the TMT on the 2-DE gel. However, the average number of protein spots on TMT gels (877) was similar to that on TMC gels (963). In the current study, we used 2-DE (18 cm×20 cm, gel size) to separate the soluble protein of human TMT, and only observed 877 protein spots on the gels stained with Coomassie brilliant blue R-350. It should be kept in mind that the limitations of 2-DE proteomics, that is, missing high molecular-weight proteins, proteins outside the pI range of the isoelectric focusing gel, many insoluble proteins, and low-abundance proteins, account for many of the TM proteins not found on the gels.

The distribution of the protein spots detected in the gel was determined by means of their pI and molecular mass (Appendix 1). According to the data measured on each gel, more than 67% of the TMT protein spots had a pI between 5 and 8, and approximately 89% of the identified spots had masses between 20 kDa and 100 kDa. Among TMC protein spots, 74% had a pI between 5 and 8, and 91% had masses between 20 and 100 kDa. In Appendix 1, there were discrepancies between the calculated pI and M_r _values, compared to the measured pI and M_r_ values for many of the identified spots. This is because we obtained the measured pI by pH gradient graphs and the measured M_r_ values by co-running molecular weight standard proteins [42]. The data accuracy was limited by reference markers and by the accuracy of measurement on the gels. The theoretical pI and M_r_ values were calculated from the amino acid sequence of a protein in the database, with fewer effects concerning the post-translation modification and the molecular stereostructure. Therefore, we could see that the many identified proteins in Appendix 1 had different measured and theoretical pI or M_r_. However, the mapping data we present here could prove to be a useful reference for future studies on differentiating expressed proteins in the TM from various glaucoma disorders.

Few early works used 2-DE to display the protein expression of human TM. Steely et al. [45] reported a human TM protein pattern obtained from 2-DE gels (16×21 cm) using radio-labeled samples visualized by X-ray film exposure. More than 400 spots were observed by this technique, but no further identification was pursued. In an earlier report, Russell and colleagues [34] compared the age-related differences of human TM protein maps on 2-DE gels. Their studies obtained approximately 160 spots from TM proteins of donors aged 9 to 91 years old. Clearly, our technique resulted in more protein spots being detected upon 2-DE gel separation. Additionally, 2-DE has been previously used to display the protein expression of TMCs with or without DEX treatment [33,46]. In the present study, we compared the 2-DE pattern of human TMT and a TMC line. We reported the pattern for 877 proteins from human normal TMT, 235 of which were identified and localized on a 2-DE gel. Since Steely et al. [33] have displayed a 2-DE protein pattern for the GTM3 cell line, and effectively identified 87 primary proteins and 93 variants of these proteins, we did not pursue detailed protein identification for those spots detected from our TMC analysis.

### Proteins identified from human trabecular meshwork

**Figure 2:**
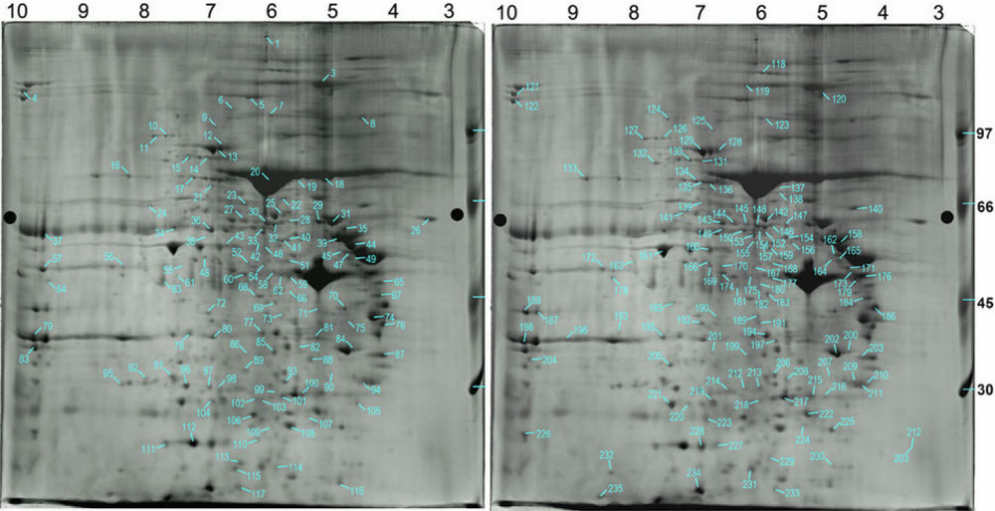
Localization of identified proteins on two-dimension electrophoresis (2-DE) gel. Two-hundred-and-thirty-five protein spots from the 2-DE gel of human TMT are localized on two images, where spots 1–117 are located on the left and 118–235 on the right. Protein spots were excised and identified by matrix-assisted laser desorption/ionization time-of-flight mass spectrometry (MALDI-TOF-MS). The spot numbers indicated on the gel images are listed in Appendix 1.

The protein spots separated on the gel (Figure 2) were identified. A total of 444 visible spots from the 2-DE gel were mechanically treated by the spot-handling workstation; 235 of these were subsequently identified by MALDI-TOF-MS. Among the 235 identified spots, 72 were determined to be identical by protein accession number. Therefore, Appendix 1 is only composed of 163 proteins with varying parameters, including protein name and accession number, theoretical and measured M_r_ and pI values, matched the peptides and amino acid sequence coverage. The spot number marked on the gel (Figure 2) for localizing a particular protein has also been indicated in Appendix 1. It was surprising to find that about one-third of the protein spots (72/235) belonged to isomers or were products of post-translational modifications. The spots representing variants of the same protein on the 2-DE of TM has previously been reported for the GTM3 cell proteome [33]. These dynamic proteins may serve as focused targets for future studies, to determine their functional significance in TM and glaucoma.

Several of the proteins we identified in TM have been reported to be associated with glaucoma. For example, myocilin was initially identified as a 55 kDa protein secreted into the media of TM cultures and induced by glucocorticoid [12,47,48]. Myocilin affects the flexibility and resilience of TM, which, if disrupted, may lead to alteration(s) in the aqueous outflow [38]. In the current study of normal human TM, myocilin was identified in two individual spots, with different molecular weights on 2-DE (spots 147 and 154 shared the same protein code, Figure 2, Appendix 1), which suggests the possibility of a post-translational modification. In addition, Steely et al. [33] suggested that calreticulin, located on the acidic side at 62 kDa (spot 26), might be modified by glycosylation, since its expected mass is 48.1 kDa. Glycosylation may influence both the calcium-signaling role and chaperonin activity of calreticulin in TMCs. Other studies have demonstrated that a mutation of cytochrome P450 (spot 54) could result in primary congenital glaucoma in some patients [17,49]. Expression of the angiopoietin-like factor CTD6, one of the 15 most abundant types of cDNA in TM [50], was believed to stimulate the deposition of specific ECM in the cornea [51]. In our present study, there were at least four spots (spot 63, 170, 181, and 182) identified as angiopoietins, which suggested this protein may play a key role in normal TM, and possibly in glaucoma. Meanwhile, several identified proteins in this study belonged to cytoskeletal protein families or extracellular matrix proteins, such as vimentin, lamin, actin, and annexin. These proteins were also the major components involved in TMCs in response to DEX or transforming growth factor β (TGF-β) treatment [7-19,29], and regulated by Rho GTPase [4]. The above-mentioned proteins are expressed in normal TMT, and they have also been reported to be changed in studies on TMCs in vitro or in vivo. We anticipate a similar scenario for other yet-uncharacterized proteins that merit future study. For example, RhoGDI (spot 225) was downregulated in our DEX-treated TMCs, which will be discussed in later sections of this paper.

The identified proteins were clustered according to gene ontology (Table 1). Based on GO biological terms, the major categories of identified proteins were metabolic process, cell adhesion, anti-apoptosis, cell motility, carbohydrate metabolic process, signal transduction, and regulation of transcription. In Table 1, we also presented the categories of identified proteins from human TMT from Bhattacharya et al. [31] and from TMCs from Steely et al. [33]. Bhattacharya et al. [31] identified 316 proteins from human TMT by SDS–PAGE and liquid chromatography (LC) tandem MS. Steely et al. [33] revealed the 2-DE based proteome of TMCs and identified fewer spots compared to our study. Generally, the major categories of identified proteins in our study were very similar to those of Bhattacharya et al. [31]. However, several kinds of proteins were absent or less abundant in the Bhattacharya et al. [31] TMT proteome. It is possible that this discrepancy is due to the the two studies’ different protein-separation techniques. We took the visible spots from 2-DE gels for the subsequent tryptic digestion and MALDI-TOF-MS analysis. In Bhattacharya et al., SDS–PAGE combined LC/MS/MS might have been able to identify some proteins present only in trace amounts.

**Table 1:** Category lists of biologic processes of TM proteins in human trabecular meshwork.

**Categories**	Present study*		**TM tissue$**		**TM cell#**	
** **	**Protein number**	**%**	**Protein number**	**%**	**Protein number**	**%**
metabolic process	19	13.4	19	6.8	4	5.6
cell adhesion	11	7.7	13	4.7	** **	** **
anti-apoptosis	11	7.7	11	3.9	6	8.5
cell motility	11	7.7	10	3.6	6	8.5
carbohydrate metabolic process	10	7.0	6	2.2	** **	** **
signal transduction	9	6.3	21	7.5	3	4.2
regulation of transcription, DNA-dependent	8	5.6	** **	** **	6	8.5
biological_process	8	5.6	13	4.7	** **	** **
nervous system development	8	5.6	5	1.8	** **	** **
glycolysis	8	5.6	14	5.0	6	8.5
transcription	7	4.9	** **	** **	7	9.9
cell cycle	7	4.9	4	1.4	4	5.6
cell proliferation	7	4.9	9	3.2	3	4.2
visual perception	7	4.9	13	4.7	** **	** **
multicellular organismal development	6	4.2	** **	** **	** **	** **
electron transport	6	4.2	7	2.5	** **	** **
ion transport	6	4.2	9	3.2	** **	** **
response to stimulus	6	4.2	6	2.2	** **	** **
response to oxidative stress	6	4.2	5	1.8	2	2.8
protein folding	6	4.2	8	2.9	12	16.9
protein targeting	6	4.2	** **	** **	** **	** **
protein amino acid phosphorylation	5	3.5	6	2.2	** **	** **
muscle contraction	5	3.5	** **	** **	** **	** **
transport	4	2.8	12	4.3	4	5.6
G-protein coupled receptor protein signaling pathway	4	2.8	5	1.8	** **	** **
proteolysis	4	2.8	10	3.6	** **	** **
cell division	4	2.8	** **	** **	2	2.8
cell-cell signaling	4	2.8	5	1.8	** **	** **
lipid metabolic process	4	2.8	10	3.6	3	4.2

The asterisk indicates the percentage was calculated on 142 proteins that matched in the cluster searching upon GO database. The dollar sign indicates data from Bhattacharya et al. [31] with 279 and the sharp (hash mark) indicated from Steely et al. [33] with 71 proteins that matched in the database.

### Dexmethasone effects on trabecular meshwork cells in vitro

**Figure 3:**
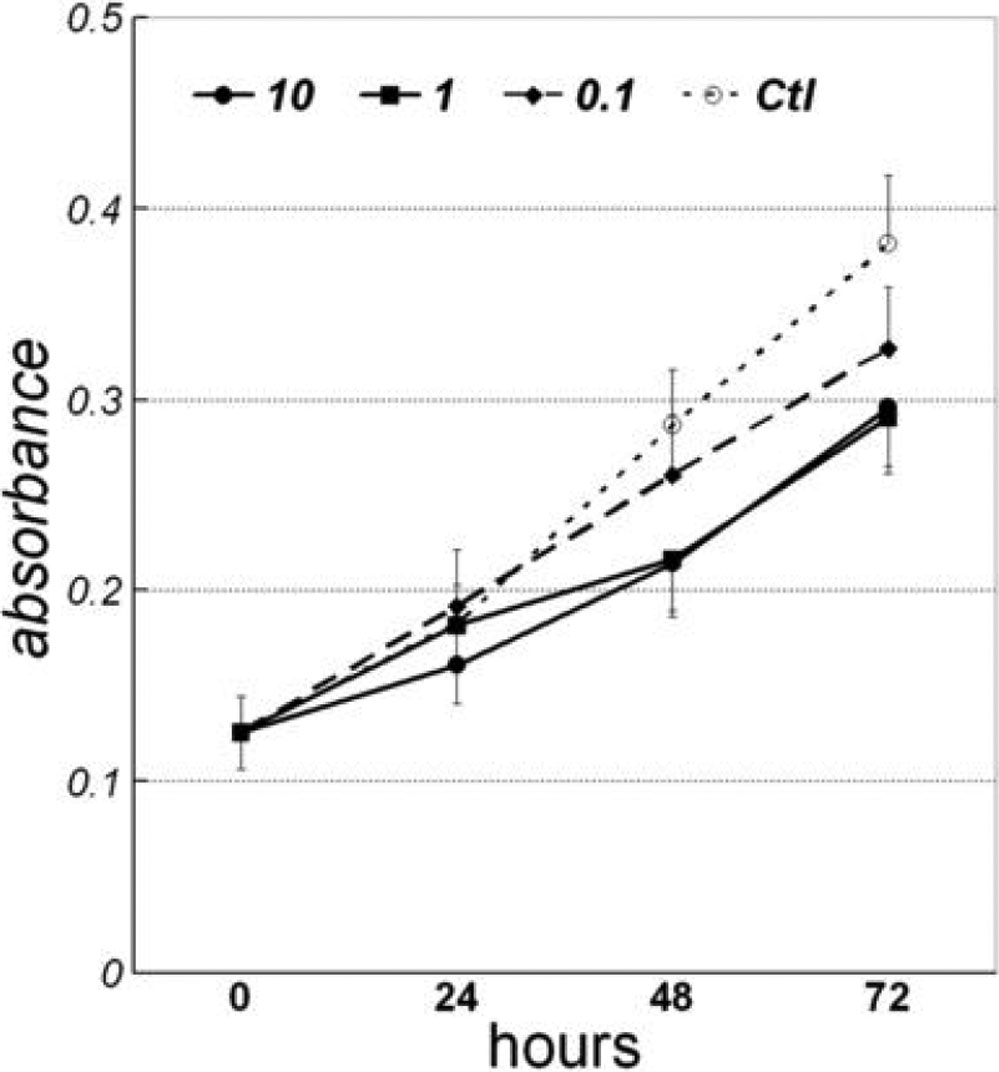
Temporal changes in the absorbance over the dexamethasone (DEX) treatment. Trabecular meshwork cells cultured in 0, 0.1, 1, and 10 μm of DEX were terminated at the indicated time, then stained with 3-(4,5-dimethylthiazol-2-yl)-2,5- diphenyltetrazolium bromide (MTT) to obtain the absorbance measurement. The data represented the results of three independent experiments. Errors on the points indicate standard deviation. Compared to control group, there were significant decreases of absorbance in 10 μm DEX-treated cells at 24 h (p<0.05) and in all three DEX groups at 48 h and 72 h (p<0.01).

We next used DEX-treated TMCs, an approach widely used to study the mechanism of glaucoma, to search for differential expression patterns of proteins. It has been reported that treating cultured TMCs for weeks with DEX causes a significant increase in average TMC size and significantly increased nuclear size [8,9]. In the present study, we could not detect significant differences between normal and DEX-treated TMCs using phase-contrast microscopy throughout the three-day study period. However, we did observe that the proliferation rate of TMCs was inhibited by 10^−5^–10^−7^ M DEX, as evidenced by MTT analysis (Figure 3). The inhibition appeared after 48 h and 72 h of DEX treatment. Generally, 10^−7^ M DEX has been widely used in previous studies [9,13,37]. Since short-term cultured TMCs were used in the current study, we used three concentrations, including two higher concentrations (10^−6^ and 10^−5^ M) of DEX, to see the more obvious effects on the cell proliferation and protein expression of TMCs. A stronger inhibition of TMC proliferation occurred at the highest DEX level. The effect of DEX occurred with the highest inhibition of cell proliferation at 10^−5^ M at 72 h of treatment.

### RhoGDI downregulated in dexamethasone-treated trabecular meshwork cells

**Figure 4:**
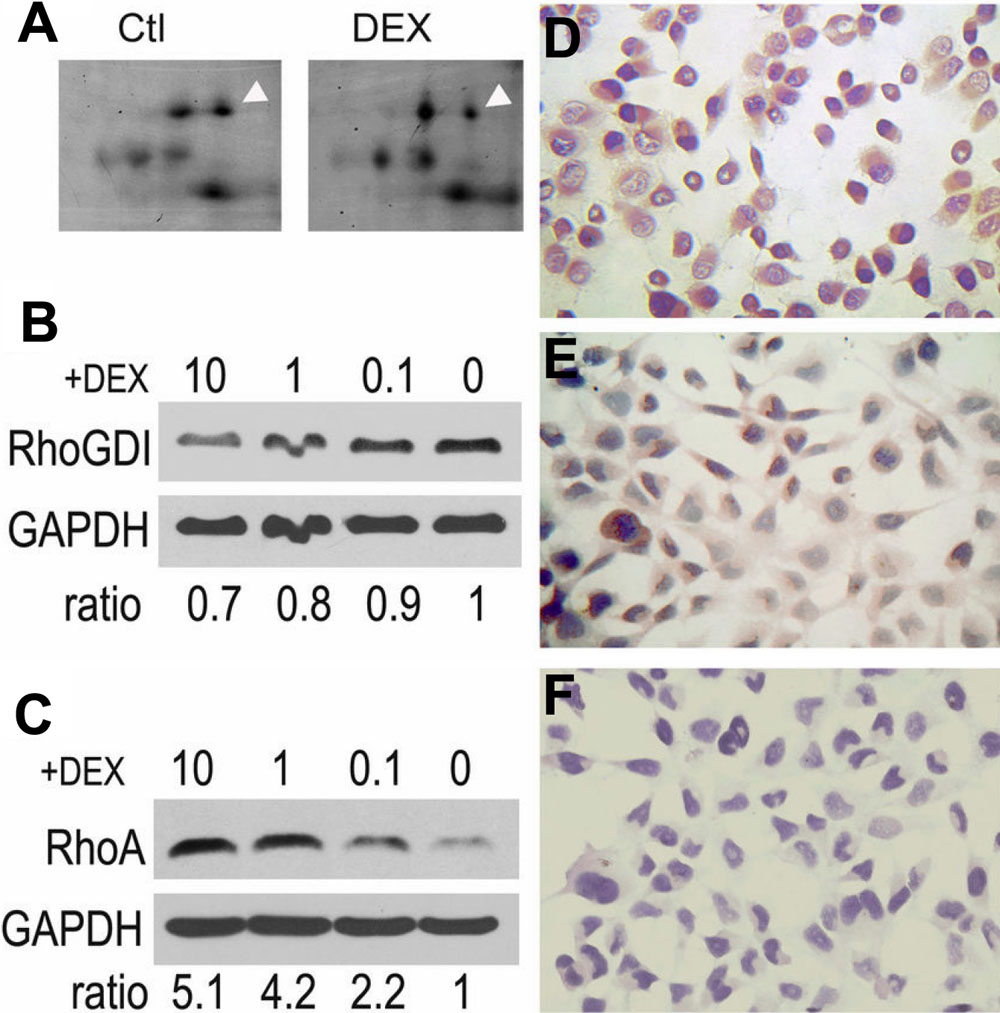
Dexmethasone-induced decreasing expression of RhoGDI and increasing expression of RhoA in human trabecular meshwork cells. **A**: 2-DE gel images showed RhoGDI spots (arrow head) of TMCs cultured with or without DEX. The spot, identified as RhoGDI by MALDI-TOF-MS, became weak in 1 μm DEX-treated TMCs. **B**: Western blotting analysis using an antibody against RhoGDI indicated the decreasing expression of RhoGDI in TMCs cultured in DEX for 48 h. **C:** The same sample in B was used to determine the expression of RhoA. A DEX-dose dependent decreasing expression of RhoA was shown in TMCs treated with DEX for 48 h. Both panel **B** and **C** western blotting analyses were repeated three to four times. The data under the western blotting photos were calculated and are shown as the average ratio of each band compared to that of the non-DEX-treated cells. Immunocytochemical staining revealed that RhoGDI displayed weaker expression in DEX-treated TMCs (**E**) than did the cells without DEX (**D**). Image **F** was a mock staining control.

We used proteomic analysis to characterize the differentially expressed proteins in cultured TMCs. We found that RhoGDI was the changed protein spot, as evidenced by immunoassays (Figure 4), which revealed that, in DEX-treated TMCs, RhoGDI expression decreased in a dose-dependent manner. By comparing the 2-DE gel patterns of the lysates from TMC lines treated with or without DEX, the RhoGDI spot exhibited lower-intensity staining (Figure 4A). By western blot analysis, the expression of the RhoGDI molecule appeared to be reduced in 24 h DEX-treated cells (Figure 4B). Higher levels of DEX displayed a general pattern of more obvious decreases, as compared to 10^−7^ M of DEX. The decrease of RhoGDI expression was also evidenced by immunocytochemical staining (Figure 4C). 10^−6^ M DEX reduced the RhoGDI staining in TM cytoplasm, compared to that in mock and control cells. Furthermore, RhoA, a target of RhoGDI, is known to function in TM as a cytoskeleton and ECM regulator. Therefore, RhoA inhibition may be the possible mechanism leading to TMC or tissue damage [4,23,52]. In the present study, we assayed the RhoA expression in our cultured cells. The results revealed that the expression of RhoA was opposite to that detected for RhoGDI by western blot assay (Figure 4D-F). It was also a dose dependent effect, with higher expression corresponding to higher DEX levels. There were a variety of reports that showed that RhoA increased the levels of promoted production of fibronectin, tenascin C, laminin, alpha-smooth muscle actin, collagen IV, laminin, and phosphorylated myosin light chains, as well as increased the expression of genes encoding ECM proteins, cytokines, integrins, cytoskeletal proteins, and signaling proteins in TMCs. The protein kinase inhibitors, including Rho kinase inhibitors, could alter TMC shape and adherence, disrupt the actin bundles, and increase aqueous outflow, thereby lowering IOP [4,53-56]. Meanwhile, RhoGDI forms a complex with the GDP-bound form of the Rho family proteins (e.g., RhoA), to regulate the activation state of RhoGTPase [26]. Thus, RhoGDI, by repressing RhoA activity, may regulate organization of the actin cytoskeleton and cell–cell adhesions in TMCs. In the present study, our results revealed that opposite effects of DEX treatment on the expression of RhoGDI and RhoA in TMCs, suggesting that DEX increases the RhoA activity, at least in part, by antagonizing the RhoGDI function. However, the detailed relationships and interactions of RhoGDI-related proteins, in glaucomatous TM in particular, remain unclear.

In summary, the current study provides 2-DE gel patterns of human TMT and a TMC line. We detected more than 850 spots for both lysates, and 235 spots were successfully identified using 2-DE gel analysis of human normal TM. These data will establish the foundation for future studies into the comprehensive protein expression patterns and effects in normal and glaucomatous TM. Furthermore, by using proteomic methods combined with immunoassays, we have provided evidence of decreasing RhoGDI expression in DEX-treated TMCs. There were distinct effects of DEX on the expression of RhoGDI and RhoA in TMCs. These results may prove useful to begin a focused study of glaucoma pathogenesis.

## Appendix 1. List of Proteins Identified on 2-DE gel.

To access the table, click or select the words “Appendix 1.” This will initiate the download of a compressed (pdf) archive that contains the file.
